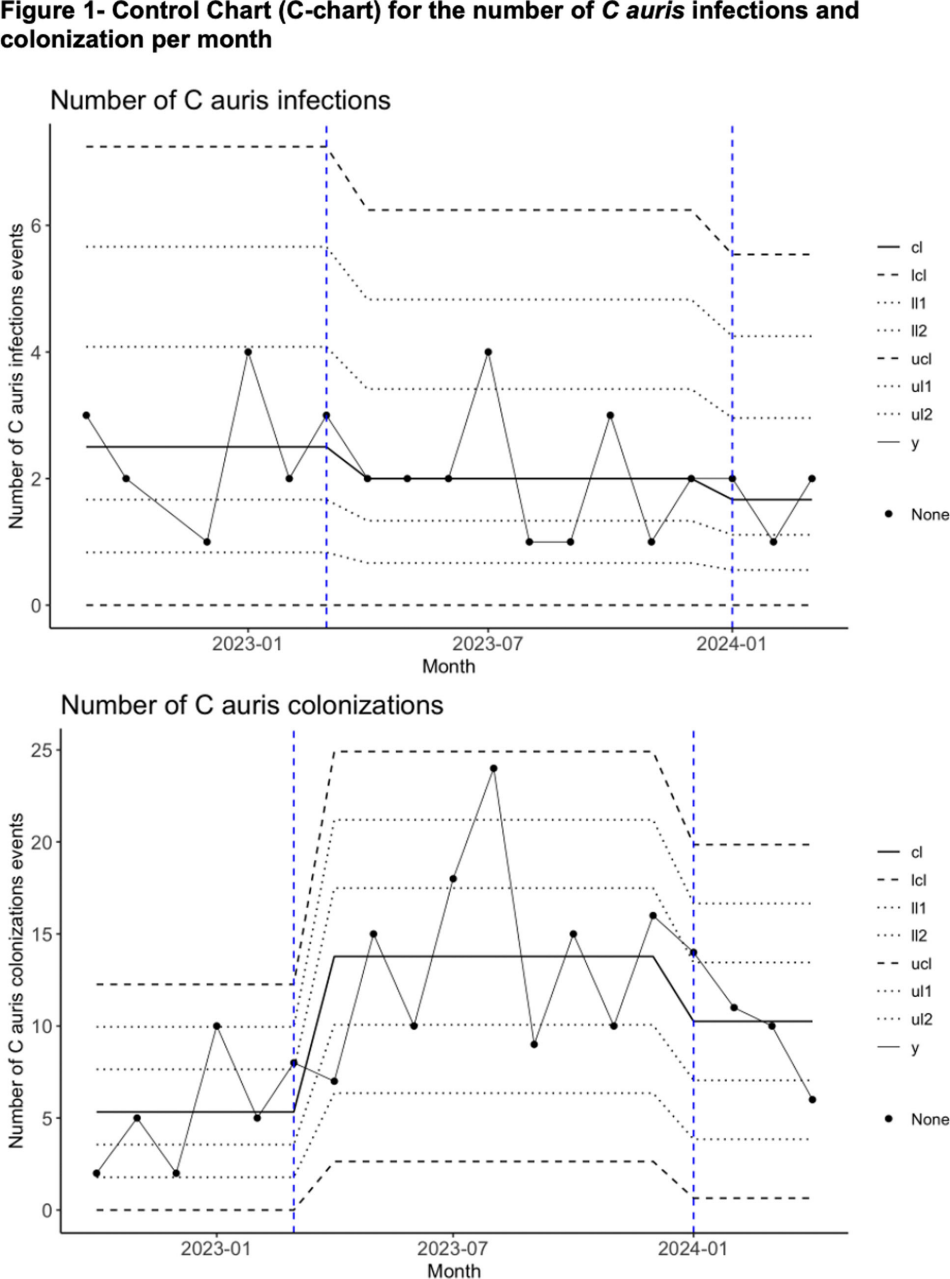# 328 Antibiotic Allergy Awareness: Insights From Hospital Passersby

**DOI:** 10.1017/ash.2026.10673

**Published:** 2026-06-23

**Authors:** Mayar Al Mohajer, Todd Lasco

**Affiliations:** 1 Baylor College of Medicine

## Abstract

**Background:** Candida auris is an emerging, healthcare?associated pathogen that persists on skin and in the environment. Early identification of colonized patients is central to interrupting transmission, but the optimal screening cadence in cluster settings is uncertain. We evaluated whether intensifying targeted screening and transitioning to in?house PCR were associated with changes in clinical cases at a large quaternary academic medical center. **Methods:** We conducted a retrospective study from November 2022 through March 2024 in hospital units experiencing C. auris clusters. Screening frequency was escalated in phases—from biweekly to weekly—and later transitioned to in?house PCR to enable faster turnaround and earlier implementation of infection?prevention actions (e.g., isolation/cohorting and enhanced environmental cleaning) per institutional protocol. The primary outcome was the monthly number of new clinical C. auris cases, defined as non?screening clinical isolates with urine and sputum excluded. The secondary outcome was colonization detected by screening PCR or by urine or sputum cultures. Monthly outcomes were monitored with statistical process control (control charts centered on phase?specific means), and effects across phases were summarized as percentage changes versus baseline. **Results:** Thirty?eight clinical cases were identified; median age was 55 years (IQR 44–65), and 66% were male. Most clinical isolates originated from blood and wound/tissue sources. Fifteen cases (39%) had a prior positive surveillance test; the median time from first positive screen to subsequent clinical isolate was 27 days (IQR 17–120). Monthly clinical cases decreased from 2.5 during biweekly screening to 2.0 with weekly screening (?20%) and to 1.7 after adoption of in?house PCR (additional ?15%; -32% overall vs baseline) (Figure 1). Colonization detections increased when screening frequency changed from biweekly to weekly (5.3 to 13.8 per month; +160%) and then declined after in?house PCR (to 10.2 per month; -26%). These patterns were concordant with control?chart signals at the phase changes. **Conclusions:** Targeted, more frequent screening coupled with rapid in?house PCR was associated with fewer clinical C. auris cases and with higher detection of colonization, consistent with earlier identification and response. Because the clinical?case metric is less sensitive to testing intensity and speciation practices than colonization counts, it may better capture the impact of hospital interventions on transmission. Findings are limited by the single?center, retrospective design, potential confounding from concurrent infection?prevention measures, and use of monthly counts rather than rates; nonetheless, the phased approach provides actionable evidence to guide surveillance strategies during C. auris clusters.

**Figure:**